# The Role of Hedgehog Signaling in Adult Lung Regeneration and Maintenance

**DOI:** 10.3390/jdb7030014

**Published:** 2019-07-09

**Authors:** Chaoqun Wang, Monica Cassandras, Tien Peng

**Affiliations:** Department of Medicine, Cardiovascular Research Institute, UCSF, San Francisco, CA 94143, USA

**Keywords:** Hedgehog signaling, asymmetric activation, adult lung regeneration, alveolar niche, emphysema, lung fibrosis

## Abstract

As a secreted morphogen, Sonic Hedgehog (SHH) determines differential cell fates, behaviors, and functions by forming a gradient of Hedgehog (Hh) activation along an axis of Hh-receptive cells during development. Despite clearly delineated roles for Hh during organ morphogenesis, whether Hh continues to regulate cell fate and behavior in the same fashion in adult organs is less understood. Adult organs, particularly barrier organs interfacing with the ambient environment, are exposed to insults that require renewal of cellular populations to maintain structural integrity. Understanding key aspects of Hh’s ability to generate an organ could translate into conceptual understanding of Hh’s ability to maintain organ homeostasis and stimulate regeneration. In this review, we will summarize the current knowledge about Hh signaling in regulating adult lung regeneration and maintenance, and discuss how alteration of Hh signaling contributes to adult lung diseases.

## 1. Hedgehog Gradient Regulates Differential Cell Fate during Development

The Hedgehog (Hh) ligands are secreted morphogens that induce concentration-specific responses along an axis of Hh-receptive cells during development [1]. The most extensively studied examples of Hh gradient are in the vertebrate limb bud and neural tube, where cells differentiate into diverse cell types depending on their proximity to the source of Hh ligands [2]. During lung development, we previously demonstrated that Hh-activated cardiopulmonary progenitor population from the venous pole of the second heart field diversifies to give rise to multiple mesenchymal lineages, and defects in Hh activation limits the repertoire of mesenchymal lineages that arises from the cardiopulmonary progenitor population [3]. These data further support the well-accepted concept that Hh generates cellular diversity through differential transcriptional output to the same stimulus. However, it is not well understood whether Hh ligands continue to form a gradient of activation in the adult tissues to maintain cellular diversity.

## 2. Asymmetric Hedgehog Activation Determines the Mesenchymal Diversity in the Adult Lung 

**Figure 1:**
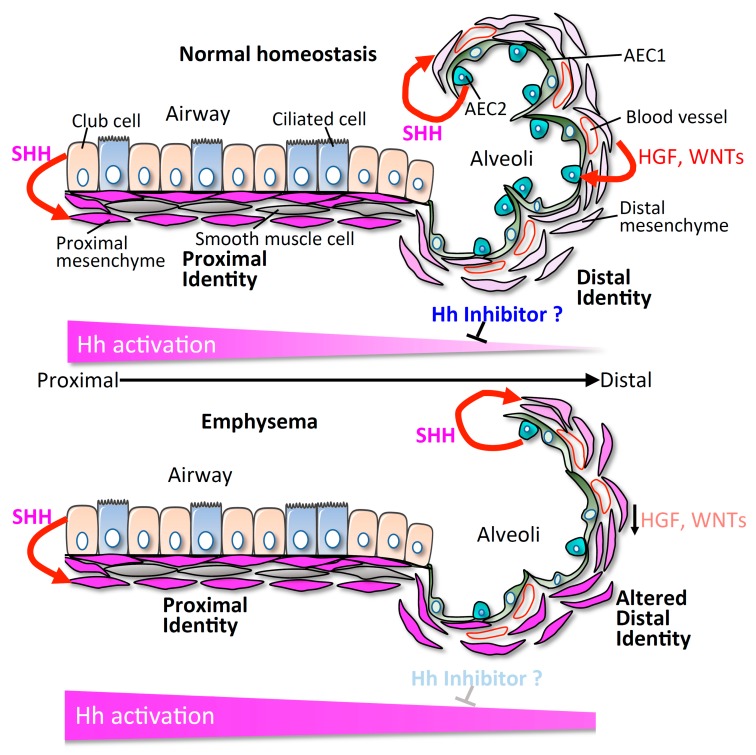
Model of asymmetric Hedgehog (Hh) activation maintaining discrete compartmental identity. We propose that differential activation of Hh is a possible mechanism in maintaining compartmental-specific identity and function in the lung. Loss of endogenous inhibitors of Hh activation could disrupt the physiological asymmetry of Hh, and lead to altered compartmental identity and structural alteration seen in lung diseases.

The adult lung is a highly subspecialized organ in terms of division of function across different compartments within the same organ (Figure 1). The lung is an air-filled organ. The trachea conducts inhaled air into the alveoli through bronchi and bronchioles (proximal airways). There are two major epithelial cell types lining the airways: ciliated cells, and secretory cells, including club cells (SCGB1A1+) [4]. The airway epithelium produces a thin surface layer of liquid containing mucins and glycoproteins to moisten the air and against pathogens. Club cells are progenitor cells that can self-renew and differentiate into ciliated cells. The airway eventually terminates and transitions into the distal alveoli, tiny air sacs composed of alveolar type 1 and type 2 epithelial cells (AEC1s and AEC2s). In adults, AEC1s cover approximately 90–95% of the alveolar surface and mediate gas exchange between the alveoli and blood [5,6]. AEC2s produce surfactant proteins to maintain alveolar fluid balance, and surfactant protein C (SFTPC) is one of the unique markers for AEC2s [7]. Furthermore, AEC2s self-renew and differentiate into AEC1s to maintain alveoli homeostasis [6,8]. The functions and behaviors of club cells and AEC2s are supported and regulated by distinct mesenchymal cell types in their respective stem cell compartments that possess the unique ability to provide feedback to their neighboring progenitor cells [9,10,11,12].

In mammals, there are three Hh ligands including Sonic (SHH), Indian (IHH) and Desert (DHH) Hedgehog [13]. SHH is the predominant ligand in the adult lung and is expressed by both the proximal airway and distal alveolar epithelium [9,10]. The GLI2+ lung mesenchymal cells are the Hh-receptive compartment, and exist in both the proximal and distal mesenchyme [9]. Surprisingly, Hh signaling activity is high in the proximal airway mesenchyme, but low in the distal alveolar mesenchyme, indicated by the enrichment of Hh target genes *Gli1*, *Ptch1*, and *Ptch2* in the proximal mesenchyme [9]. Based on the differential gene expression, the proximal airway mesenchyme and the distal alveolar mesenchyme can be clustered into two distinct cell populations. We found that the asymmetry of Hh activation promotes this anatomical segregation between the proximal and distal mesenchyme by promoting proximal signature genes and suppressing the distal signature genes, which determines distinct airway and alveolar progenitor niches [9]. 

## 3. Active Hedgehog Activation Keeps Airway Quiescence

Normal adult tissue quiescence is thought to be a default state in the absence of a proliferative stimulus such as injury. Hh activation is typically regarded as a mitogenic cue during tissue development, injury/repair and tumorigenesis [14]. A previous study has shown that Hh signaling activity is absent in the adult airways, indicated by negative immunohistochemical detection of SHH and GLI1 [15]. Depletion of club cells by naphthalene administration induced airway repair/regeneration, which was accompanied by upregulation of SHH and GLI1 in the epithelial compartment [15]. These results suggest that Hh signaling promotes cell proliferation during lung injury/repair. In contrast, with multiple genetic mouse models, we found that Hh signaling continues to be active during normal homeostasis to maintain quiescence in the lung [10]. With the *Gli1^LacZ/+^* reporter mice, Hh-active cells (GLI1+) are predominantly present around airways and proximal vessels and are less seen in the alveolar interstitium [16]. Lineage labeling in adult lung showed that the GLI1+ cells are mesenchymal cells, expressing several mesenchymal markers including Pdgfrα, Pdgfrβ, Col1a1, and Vimentin [10]. Conditional deletion of *Shh* in the SCGB1A1+ club cells induced mesenchymal proliferation. Consistently, deletion of the Hh effector *Smo* in the proximal mesenchymal cells also resulted in mesenchymal expansion. Furthermore, inactivation of Hh signaling in the mesenchyme further promoted the cell proliferation of the airway Club cells, suggesting a role for SHH in regulating epithelial turnover in a non-cell autonomous manner. During the injury–repair process, ablation of the club cells by naphthalene injection led to downregulation of Hh signaling, indicated by reduced expression of *Shh* and *Gli1*, and loss of mesenchymal quiescence, which in turn stimulates epithelial regeneration to replete the airway club cells until homeostasis is re-established. All of these findings suggest that the lung airway epithelial cells keep the proximal mesenchymal quiescence through paracrine Hh signaling, which also regulates a feedback loop to maintain epithelial quiescence. However, the downstream target genes of Hh signaling in the proximal mesenchyme remain unknown, and we are still unclear about how the Hh-active mesenchyme maintains airway epithelial quiescence. Hh activation in the lung mesenchyme *in vitro* suppresses the epithelial mitogens including HGF and WNTs [9], which might be one of the mechanisms for how Hh signaling keeps airway quiescence *in vivo*.

## 4. Alveolar Mesenchyme Supports the Regeneration of Alveolar Progenitors

Generation of the alveolus requires intricate interactions between multiple cell lineages [17]. In mice, alveologenesis initiates after birth. At birth, the alveolar region is composed of saccules [17]. Most of the surface of the saccules are covered by AEC1s, and the remaining surface is filled in by AEC2s [17,18]. The drastic expansion of alveolar surface area occurs at about postnatal (P) day 5, and continues until P30 [18]. In humans, some alveoli have formed before birth, and the process of alveologenesis continues into adolescence [17,19]. Alveologenesis requires the proliferation and differentiation of epithelial progenitors by coordinating with the mesenchyme. GLI1+ mesenchymal cells are present in both the airway and alveoli during the first week of postnatal life, and then the number of alveolar GLI1+ cells decreases gradually during alveologenesis [16], suggesting that dynamic regulation of the Hh activation domain is required for the transition from alveologenesis to alveolar maintenance. The presence of Hh activation in the alveoli is likely important during the alveolarization process, as inhibition of Hh signaling with anti-SHH mAb during early alveologenesis disrupted the formation of alveoli by inhibiting GLI1+ cell differentiation into alveolar myofibroblasts [16,20]. However, what is not known is whether the physiologic regression of Hh activation from the alveolar domain is also necessary for proper alveolar maintenance after alveolarization is complete. These results suggest that tight regulation of Hh signaling in alveolar mesenchyme is critical for alveolar formation, and sophisticated mouse genetic tools might be needed to further clarify the role of Hh signaling during alveologenesis.

In adults, alveolar homeostasis is maintained by the alveolar progenitor AEC2s. The functions of AEC2s are supported by the adjacent alveolar mesenchyme during adult alveolar maintenance. With the 3D mesenchyme-AEC2 co-culture system, it has been shown that growth and differentiation of the AEC2-derived organoids occurred most readily when co-cultured with alveolar mesenchyme [8,11,19], suggesting that alveolar mesenchyme contributes to alveolar maintenance and regeneration by promoting both the proliferation and differentiation of AEC2s. Previous studies have shown that PDGFRα+ mesenchymal fibroblasts are necessary for alveolar organoid development *in vitro* [8]. The PDGFRα+ cells are present both in the airway and alveoli, and contain many different lineages including WNT2+ and AXIN2+ mesenchymal cells. Further studies showed that AXIN2+/PDGFRα+ cells are preferentially capable of promoting the self-renewal and differentiation of AEC2s, compared with AXIN2+, WNT2+, PDGFRα+, and other lineage negative mesenchymal cells [11,21]. We have recently shown that the majority of the proximal airway GLI2+/GLI1+ and alveolar GLI2+/GLI1− mesenchymal cells are also PDGFRα+ [9]. The alveolar GLI2+/GLI1− mesenchyme with low Hh activation expresses high levels of HGF and WNT2, and activation of HGF and WNT signaling pathways promote the growth of AEC2s *in vitro* [9]. Expansion of Hh activation in the alveolar mesenchyme by inducing the expression of a constitutively active form of the Hh effector, *Smo* (SmoM2) [22], in the entire Hh-competent GLI2+ mesenchyme inhibits the regeneration of AEC2s by reducing the expression of HGF and WNTs, resulting in a loss of alveoli and enlargement of airspace, typical characteristics of emphysema [9]. These results suggest that restriction of Hh activation in the alveolar mesenchyme is important for maintaining alveolar regeneration, and hyperactivation of Hh signaling can cause emphysema.

## 5. Hedgehog-Interacting Protein (*HHIP*) Is Implicated in COPD/Emphysema

Chronic obstructive pulmonary disease (COPD) is currently the fourth leading cause of death worldwide according to the World Health Organization [23]. COPD is defined as a progressive pulmonary disease that makes it difficult to breathe. Emphysema, a major subtype of COPD, is characterized by loss of alveoli. Cigarette smoke (CS) exposure is the greatest risk factor for the development of emphysema [24]. However, only certain individuals exposed to CS are susceptible to developing emphysema, suggesting that individual differences in genetic background are likely to be an important determinant [25,26,27]. Genome-wide association studies (GWAS) in several human cohorts have consistently identified susceptibility locus for COPD/emphysema on chromosome 4q31 adjacent to hedgehog-interacting protein (HHIP) gene [26,28,29,30]. Genomic analysis of the 4q31 locus suggests possible enhancer elements that regulate the expression of *HHIP* [31]. Furthermore, reduced *HHIP* expression was observed in emphysema tissues [32], suggesting a protective role of HHIP in emphysema pathogenesis. Using the *Hhip* heterozygous mice (*Hhip^LacZ/+^*), previous studies have shown that this *Hhip* haploinsufficiency exaggerated CS-induced airspace enlargement with increased lymphoid aggregates, and enhanced lymphocyte activation pathways in the lungs, suggesting that dysregulated immune responses in *Hhip* heterozygous mice associated with the severity of airspace enlargement [33]. Further studies showed that *Hhip^LacZ/+^* mice developed spontaneous emphysema and lung function impairment over 10 months. This age-related emphysema is associated with increased oxidative stress levels in the lungs of *Hhip* heterozygous mice, and can be improved by treating the mice with antioxidant *N*-acetyl cysteine [34].

HHIP is a negative regulator of Hh signaling. HHIP inhibits Hh signaling by acting as a decoy receptor for SHH [35]. As a transmembrane protein, HHIP is almost entirely extracellular with no known downstream signaling capacity in the cytoplasm [36]. HHIP is vertebrate-specific, suggesting that vertebrates may have evolved novel mechanisms of Hh modulation, that could facilitate distinct aspects of vertebrate development and organ maintenance. During early lung development, loss of HHIP increases Hh activation, resulting in defective branching morphogenesis [37]. HHIP expression also persists in human adult tissues, with the highest expression in the lung (www.proteinatlas.org), suggesting that it plays an important role in maintaining adult lung homeostasis. By analyzing the transcriptome profiles of COPD/emphysema patients in the largest study to date that correlated gene expression within epithelial brushings with clinical disease severity, we found a significantly positive correlation between *SHH* expression in the epithelium, and the disease severity as measured by FEV1, suggesting that hyperactivation of Hh signaling could be one of the pathogenic mechanisms of COPD/emphysema. Indeed, previous studies showed that lung cells from human COPD/emphysema patients exhibited Hh hyperactivation with upregulated GLI1 and PTCH1, compared with those from non-COPD/emphysema controls [38,39]. However, there is still a lack of studies that examine the association between hedgehog activation and HHIP-related emphysema. We have recently shown that asymmetric Hh activation in the mesenchyme is crucial in maintaining alveolar progenitor cell niche [9], suggesting that HHIP might regulate the function of the alveolar mesenchyme by restricting Hh signaling.

## 6. Hedgehog Is Dysregulated in Pulmonary Fibrosis

Idiopathic Pulmonary Fibrosis (IPF) is a progressive disease characterized by loss of alveolar architecture and inefficient gas exchange due to excessive scarring [40]. IPF lungs are characterized by the presence of myofibroblasts, accompanied by the accumulation of extracellular matrix that results in fibrotic scarring [41]. Recent studies have reported dysregulation of various developmental pathways, including Hh, in IPF patients. Increased *SHH* expression has been shown in alveolar epithelial cells lining honeycomb cysts in IPF patients [42,43,44]. Components of the Hh pathway, including *SHH*, *PTCH1*, *SMO*, *GLI1*, and *GLI2*, are upregulated in IPF lungs [45,46]. In murine models, GLI1+ cells have been shown to expand and give rise to myofibroblasts after bleomycin-induced lung fibrosis [10,47], and conditional inhibition of Hh signaling in Collagen I-expressing mesenchymal cells reduced myofibroblast differentiation and collagen content [46]. However, therapeutic blockade of the Hh pathway has produced mixed results, as demonstrated by studies showing lack of efficacy using antibody inhibition of Hh signaling and SMO pharmacologic blockade in bleomycin-induced lung fibrosis [16,48]. While pharmacologic targeting of GLI was effective in reducing collagen accumulation as a primary endpoint [48], genetic ablation of *Gli1* failed to replicate the effect of the pharmacologic blockade [49]. These results suggest that Hh signaling has a more nuanced role in modifying the fibrotic response to epithelial injury, which may not be directly related to the accumulation of collagen in these murine studies of lung fibrosis.

## 7. Conclusions and Future Directions

A popular concept in regenerative biology is that developmental pathways “re-emerge” during injury repair to recapitulate developmental processes and reconstruct the organ. Our perspective, based on newer work, is that certain developmental pathways are never turned off, but repurposed to maintain cellular homeostasis and organ function. In the case of Hh in the lung, the activity of the pathway never fully disappears from development to adulthood, but the domain of activation shifts dramatically across the different epochs of development. This property allows Hh to generate differential responses across anatomical compartments within the lung to impart a distinct cellular identity and behavior (Figure 1). Physiological or genetic susceptibilities that alter the Hh activation domains can then disrupt normal homeostatic cellular processes, and disrupt organ architecture, as seen in the cases of emphysema and lung fibrosis. These findings lead to more questions about how asymmetric Hh activation domains are maintained and regulated in the adult lungs, including: 1) How does the Hh ligand gets transported from the epithelium to the mesenchyme in different anatomical segments of the lung? 2) Do non-Hh responder cells play a role in modifying the asymmetric activation of Hh in the lung? 3). Does alterations of Hh activation domain occur in other chronic lung diseases? The major current knowledge gaps and directions to approach these gaps are summarized in Table 1. A more nuanced understanding of the role of Hh in these physiological processes could lead to more rational ways to target the pathway in adult lung diseases.

## Figures and Tables

**Table 1:** Current knowledge gaps and future directions on Hh in adult lung.

Current Knowledge Gaps	Future Directions
How do non-Hh responsive cells in the lung shape the differential activation of Hh?	Single cell analysis of the lung to examine the spatial distribution of factors that might modify Hh signaling.
How does the Hh ligand get transported from the epithelium to the mesenchyme?	Closer examination of cellular features such as the primary cilia or possibly cytonemes that have been implicated in Hh transduction.
Does the alteration of Hh activation domain occur in other disease contexts?	Examination of how Hh activation domains shifts in other disease such as lung fibrosis and infection.
